# Heterogeneous Reaction of SO_2_ on Manganese Oxides: the Effect of Crystal Structure and Relative Humidity

**DOI:** 10.1038/s41598-017-04551-6

**Published:** 2017-07-03

**Authors:** Weiwei Yang, Jianghao Zhang, Qingxin Ma, Yan Zhao, Yongchun Liu, Hong He

**Affiliations:** 1 State Key Joint Laboratory of Environment Simulation and Pollution Control, Research Center for Eco-Environmental Sciences, Chinese Academy of Sciences, Beijing, 100085 China; 2 College of Resources and Environment, University of Chinese Academy of Sciences, Beijing, 100049 China; 3 Center for Excellence in Urban Atmospheric Environment, Institute of Urban Environment, Chinese Academy of Sciences, Xiamen, 361021 China; 4 Washington State University, 1505 Stadium Way, Pullman, Washington State 99164 USA

## Abstract

Manganese oxides from anthropogenic sources can promote the formation of sulfate through catalytic oxidation of SO_2_. In this study, the kinetics of SO_2_ reactions on MnO_2_ with different morphologies (α, β, γ and δ) was investigated using flow tube reactor and *in situ* Diffuse Reflectance Infrared Fourier Transform Spectroscopy (DRIFTS). Under dry conditions, the reactivity towards SO_2_ uptake was highest on δ-MnO_2_ but lowest on β-MnO_2_, with a geometric uptake coefficient (γ_obs_) of (2.42 ± 0.13) ×10^–2^ and a corrected uptake coefficient (γ_c_) of (1.48 ± 0.21) ×10^−6^ for the former while γ_obs_ of (3.35 ± 0.43) ×10^−3^ and γ_c_ of (7.46 ± 2.97) ×10^−7^ for the latter. Under wet conditions, the presence of water altered the chemical form of sulfate and was in favor for the heterogeneous oxidation of SO_2_. The maximum sulfate formation rate was reached at 25% RH and 45% for δ-MnO_2_ and γ-MnO_2_, respectively, possibly due to their different crystal structures. The results suggest that morphologies and RH are important factors influencing the heterogeneous reaction of SO_2_ on mineral aerosols, and that aqueous oxidation process involving transition metals of Mn might be a potential important pathway for SO_2_ oxidation in the atmosphere.

## Introduction

Sulfate species contribute substantially to tropospheric aerosols, with a significant cooling effect on the global climate by scattering solar radiation and acting as cloud condensation nuclei (CCN)^1^. In addition, sulfate has been reported to play a significant role in the haze formation in China in recent years^2–4^. There are a variety of formation routes for sulfate aerosols, such as, direct evolution of H_2_SO_4_ and oxidation of sulfur-containing gas^5–7^. SO_2_ is the predominant sulfur-containing atmospheric gas, which is released into the troposphere mainly by fossil fuel combustion and volcanic emission. The conversion of SO_2_ to sulfate aerosols can proceed in several ways, including gas-phase oxidation of SO_2_ by OH radical and aqueous-oxidation by H_2_O_2_, O_3_ in cloud water and fog droplets^5, 8^. Recently, Wang *et al*.^9^ have proposed a new aqueous-oxidation pathway for sulfate aerosols formation, in which NO_2_ in cloud droplets or on aerosol water contributed considerably to the oxidation of SO_2_ by high concentration of NH_3_ neutralization, exacerbating severe haze development. However, these sources are still not sufficient to explain the discrepancy between field measurements and modeling results for sulfate formation, and that SO_2_ oxidation tends to be underestimated in winter source regions lacking cloud or fog, mostly in outbreak areas of haze, suggesting missing oxidation mechanisms of SO_2_ in the atmosphere^4, 10–13^.

As one of the most important aerosols in mass terms, mineral dust entrained into the atmospheric can interact with atmospheric trace gases in the presence of sunlight or water, such as by providing reactive surfaces in the heterogeneous uptake of SO_2_^12, 14–16^. Early research found that conversion of SO_2_ to sulfate species was closely associated with mineral dust, accounting for 50–70% of aerosol sulfate in the vicinity of the dust source regions^17^. Moreover, this positive correlation seemed to show an important role in the haze formation occurring in China in recent years^12^. During the past decades, heterogeneous reactions of SO_2_ on sea salts^18^, soot^19, 20^, CaCO_3_^21–23^, metal oxides^12, 24–29^ and authentic dust^30, 31^ have been widely investigated. A recent study found that metal oxides present in mineral dust induced photocatalytic reaction of SO_2_ to sulfate^3^. Harris *et al*.^32^ reported that sulfate formation was dominated by catalytic oxidation of SO_2_ by natural transition metal ions on coarse mineral dust. Thus the catalytic oxidation of SO_2_ to sulfate initiated by transition metals cannot be neglected.

Transition metal ions, i.e., Mn(II) and Fe(III) were found present common in dust particles and lead to significantly catalytic oxidation of S(IV) with dissolved oxygen in aqueous phase^5, 33^. For iron-containing dust, a number of studies are available investigating the influences of its morphology and existing water on the heterogeneous oxidation of SO_2_^25, 29, 34–36^. For instance, Fu *et al*.^34^ reported that α-Fe_2_O_3_ achieved the best performance in the catalytic oxidation of SO_2_ among different crystal phases of iron oxides. In particular, a recent inclusion of parameterization into models simulation involving Fe^3+^-catalyzed SO_2_ heterogeneous oxidation in aerosol water successfully reproduced the rapid sulfate growth during haze days in China^11^. Both iron and relative humidity played key roles in promoting the uptake of SO_2_ to aerosol surface, with a high reactive uptake coefficient of 0.5 × 10^−4^ assuming enough alkalinity in the catalytic reaction^11^. In the atmosphere, the presence of trace manganese oxide derived from mineral dust, fossil fuel deposits, fuel-oil fly ash, metal processing industry, etc. may also have a significant effect on the SO_2_ oxidation rate through a redox chemistry process^19, 37^. However, up to now, no study has investigated the influence of its crystalline forms on the oxidation of SO_2_, though a few studies involving the effect of the phase structure of manganese oxides on the catalytic oxidation of CO and HCHO have appeared, let alone the influence of water under ambient conditions^38, 39^. Thus, in the present study, we investigated the effect of MnO_2_ crystalline form on the reactivity of SO_2_ oxidation and the influence of water during this process using a flow tube reactor and DRIFTS. The results could help understand the role of Mn in the heterogeneous formation of sulfate.

## Results and Discussion

### Structures and morphologies

**Figure 1:**
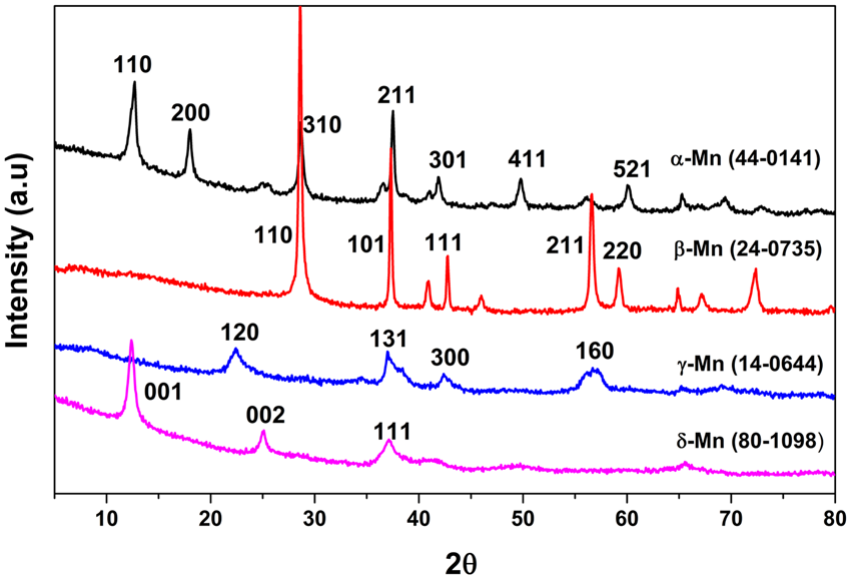
XRD patterns of α-, β-, γ- and δ-MnO_2_ samples.

Figure 1 showed the XRD profiles of the MnO_2_ samples. The diffraction peaks of these MnO_2_ samples matched well with standard patterns of α-MnO_2_ (JCPDS 44-0141), β-MnO_2_ (JCPDS 24-0735), γ-MnO_2_ (JCPDS 14-0644) and δ-MnO_2_ (JCPDS 80-1098). It was found that γ- and δ-MnO_2_ displayed poor crystallinity compared with those of α- and β-MnO_2_ due to their disordered structures in certain crystallographic directions^38, 40^. The average sizes of α-, β-, γ- and δ-MnO_2_ were 25.98, 25.75, 17.79 and 12.39 nm as calculated using the Scherrer equation as listed in Table 1.

**Table 1:** Summary of physical properties and SO_2_ uptake capacities and uptake coefficients for the heterogeneous reaction of SO_2_ on manganese oxides.

Sample	BET area (m^2^ g^−1^)	Pore volume (cm^3^ g^−1^)	Average particle size^a^ (nm)	SO_2_ uptake capacity		Uptake coefficient, γ	
				(×10^−3^ g g^−1^)	(**×**10^17^ molecules m^−2^)	γ_obs, initial_ (**×**10^−3^)	γ_c, initial_ (10^−6^)
α-MnO_2_	80.8	0.27	25.98	1.30 ± 0.11	1.51 ± 0.13	7.07 ± 0.72	0.68 ± 0.34
β-MnO_2_	23.3	0.05	25.75	0.94 ± 0.09	3.79 ± 0.36	3.35 ± 0.43	0.74 ± 0.30
γ-MnO_2_	85.3	0.26	17.79	3.28 ± 0.29	3.61 ± 0.32	13.2 ± 1.39	0.78 ± 0.25
δ-MnO_2_	108.4	0.38	12.39	18.83 ± 1.01	16.34 ± 0.88	24.2 ± 1.31	1.48 ± 0.21

^a^Average size calculated using Scherrer equation derived from XRD measurement.

**Figure 2:**
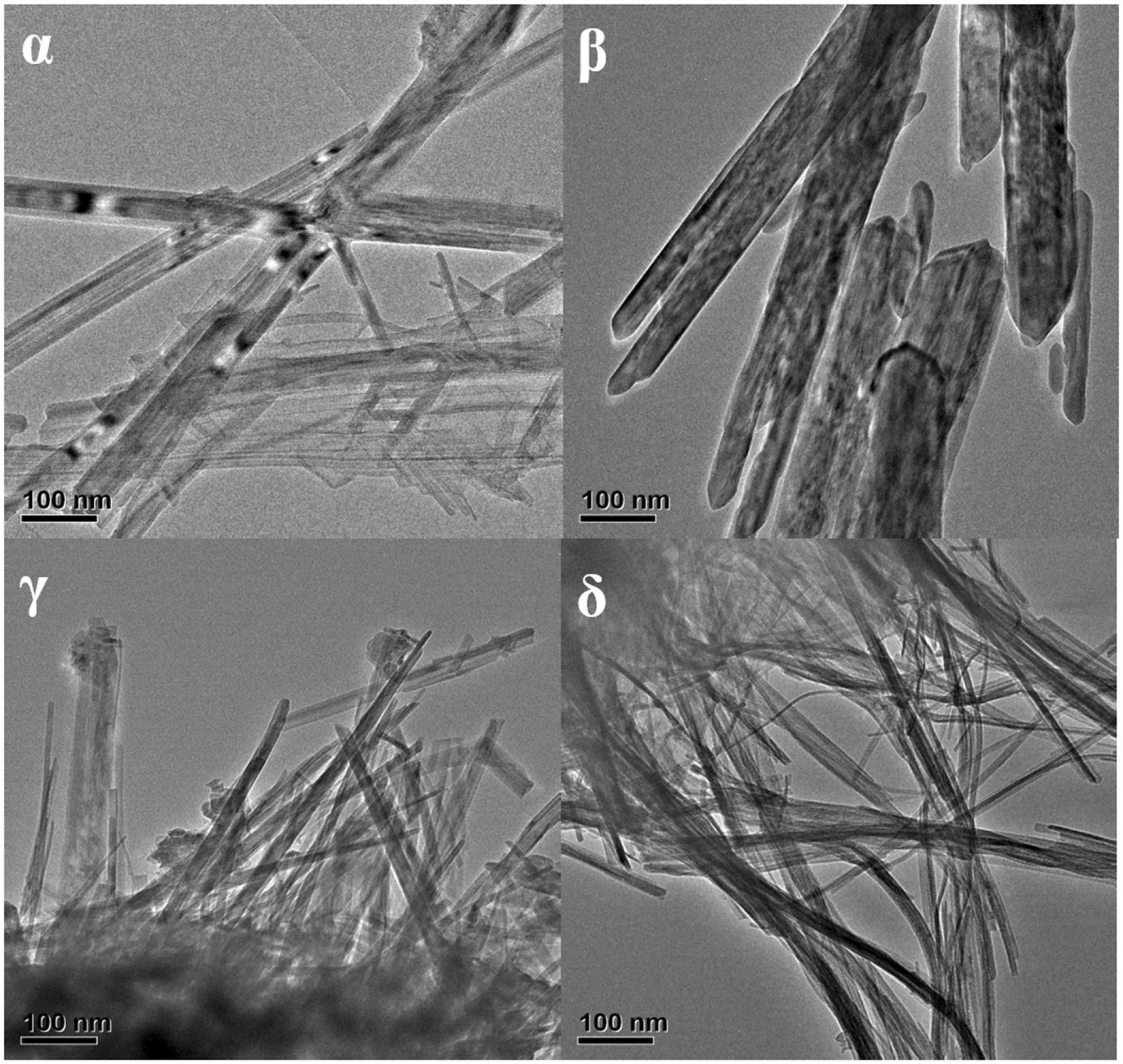
TEM images of α-, β-, γ- and δ-MnO_2_.

The morphologies of the different crystalline manganese oxides were investigated using FE-SEM (Fig. S1) and HR-TEM (Fig. 2). Detailed description of the data was reported in our previous study^38^. Both α- and β-MnO_2_ showed dendritic nanostructures consisting of nanorods (Fig. S1); the former were 40–80 nm wide and 2.5 μm long whereas the latter were 50–100 nm wide and 1 μm long (Fig. 2). In contrast, γ- and δ-MnO_2_ had a similar spherical morphology composed of nanowires ranging 10–20 nm in diameter. It was noted that the nanostructure for δ-MnO_2_ consisted of very thin and long nanofibers compared to the short nanoneedles observed for γ-MnO_2_. Since the manganese oxide varied in structure and morphology, their oxidation activity towards SO_2_ should be different, and thus the reactions under different conditions were investigated as discussed below.

### Reaction under dry conditions

**Figure 3:**
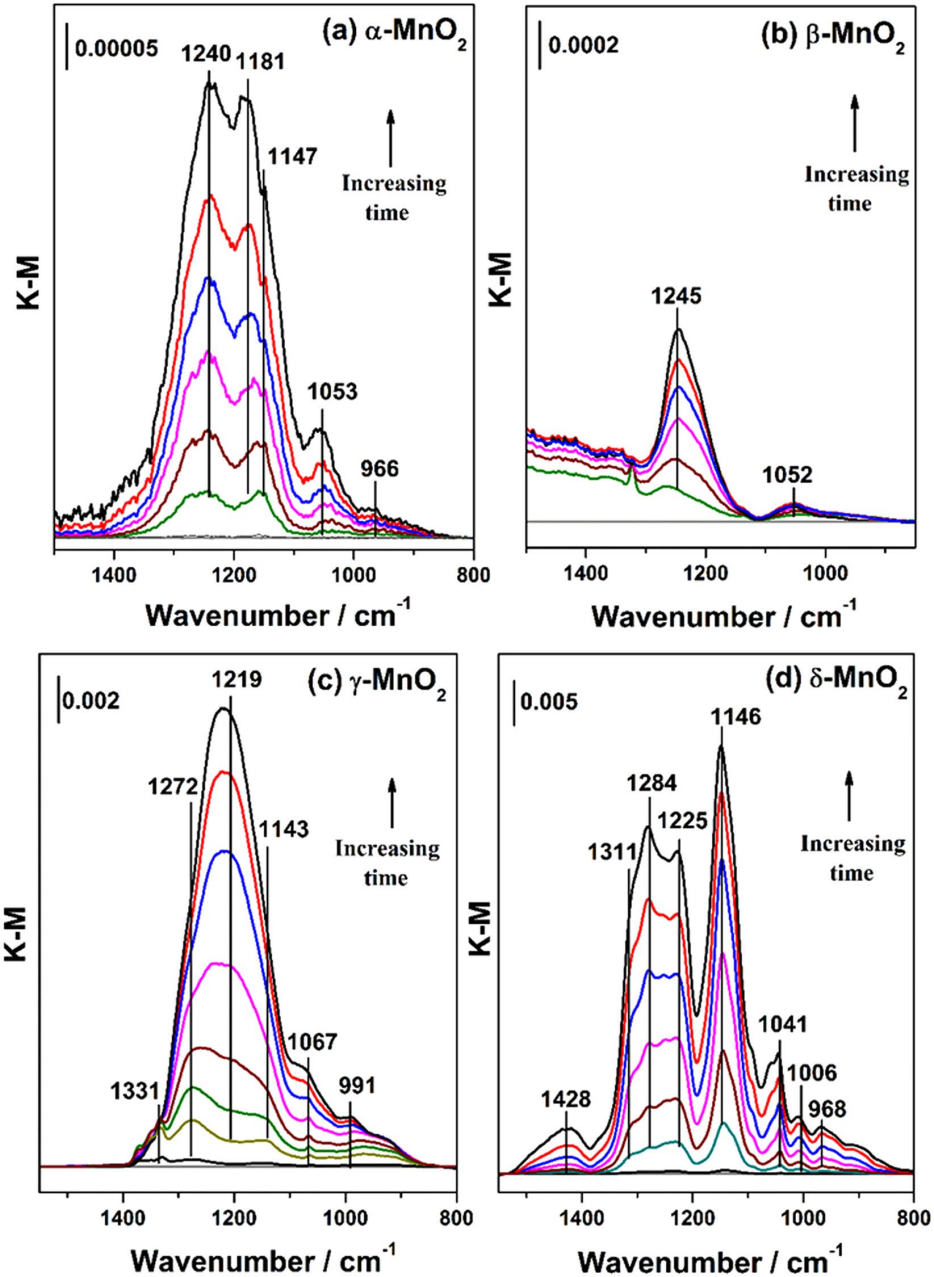
DRIFTS spectra recorded for the heterogeneous reactions of 40 ppmv SO_2_ on (**a**) α-, (**b**) β-, (**c**) γ-, (**d**) δ-MnO_2_ as a function of time under dry conditions, balanced with synthetic air in a total flow of 100 mL/min. The reaction time was 60 min.

Figure 3 showed the DRIFTS spectra of MnO_2_ exposed to SO_2_ under dry conditions as a function of time. The relationships between the coordination modes of sulfate complexes and their infrared vibrational bands have been well established^41^. There are two infrared sulfate vibrations, i.e., nondegenerate symmetric stretching ν_1_ and triply degenerate asymmetric stretching ν_3_. A free sulfate species is tetrahedral (T_d_ symmetry), only having one triply degenerate band at 1100 cm^−1^ ^42^. When a monodentate surface complex forms by bonding of one oxygen atom (C_2ν_), the ν_3_ mode splits into two bands, one above 1100 cm^−1^ and one lower than 1100 cm^−1^, while the ν_1_ mode becomes active at around 975 cm^−1^. In the case of a bidentate structure, the ν_3_ band splits into more than two bands in the region of 1000–1250 cm^−1^ in addition to the infrared active band of the ν_1_ mode at 975 cm^−1^ ^43^.

It was observed that the adsorption of SO_2_ on the four crystal manganese oxides was different. For α-MnO_2_, five weak bands assigned to bidentate sulfate species appeared at 1240, 1181, 1147, 1053 and 966 cm^−1^ ^44^. Similarly, SO_2_ interacted with β-MnO_2_ weakly and only two bands at 1245 and 1052 cm^−1^ were observed, which was likely due to an outer-sphere surface complex formed by electrostatic attraction, with the minimal distortion from T_d_ symmetry in this case^34^.

Compared to α- and β-MnO_2_, strong adsorption of SO_2_ on γ- and δ-MnO_2_ occurred. The γ-MnO_2_ sample showed somewhat similar spectral characteristics to those of α-MnO_2_ except for blue-shift of bands to higher frequencies at 1272, 1219, 1143, 1067 and 991 cm^−1^, indicating a closer interaction between SO_2_ and γ-MnO_2_^42^. In addition, the presence of a band at 1331 cm^−1^ suggested that sulfate species accumulate on the surface^45^. The reaction of SO_2_ on δ-MnO_2_ may follow a different principle because a great number of bands attributed to sulfate species grew in intensity upon adsorption of SO_2_, mostly in the higher vibrational region of 1450–1250 cm^−1^. The results indicated that polymeric sulfate species may dominate on the surface of δ-MnO_2_^46^.

**Figure 4:**
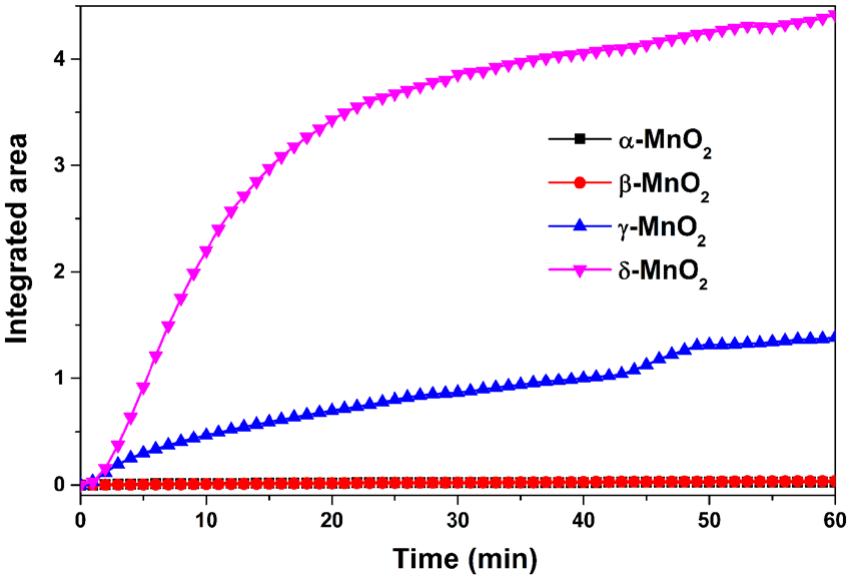
Comparison of integrated areas ranging 1552–782 cm^−1^ for the sulfate species formed on different-crystal manganese oxides.

To compare the amounts of sulfate formed on the surfaces of manganese oxides, the integrated areas associated with related bands for α-, β-, γ- and δ-MnO_2_ were shown in Fig. 4. It was found that the amount of sulfate formed grew linearly with time at the initial stage. Then the reaction rate slowed down until the surface was almost saturated with sulfate. The oxidation reactivity of SO_2_ on manganese oxides decreased in the order of δ- > γ- > α- ≈ β-MnO_2_. Since DRIFTS spectra only gave the amount of sulfate formed on the surface, further study regarding the uptake of SO_2_ was conducted in the flow tube reactor.

**Figure 5:**
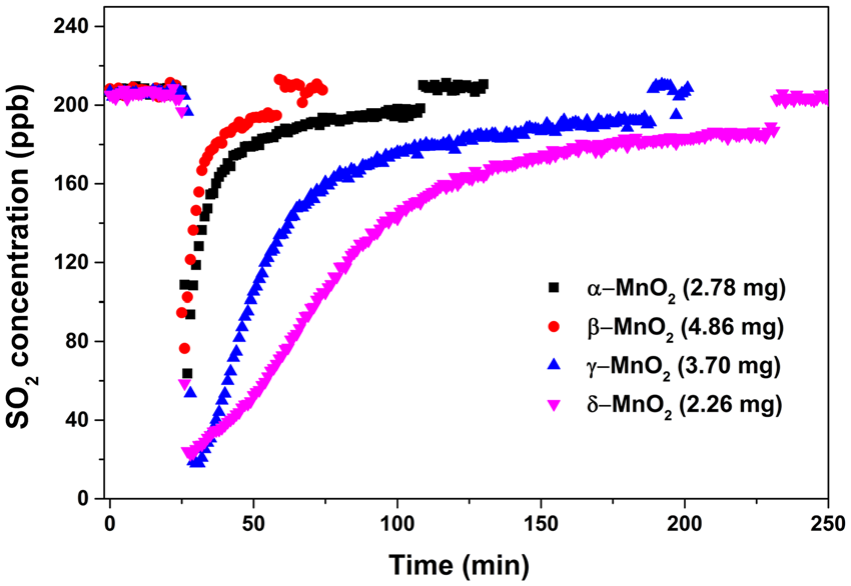
Uptake curves of SO_2_ on different crystal manganese oxides.

In the uptake experiments, a series of SO_2_ uptake curves were obtained for different manganese oxides. It was noted that the concentration of SO_2_ cannot reach the initial state completely but only approached it continuously due to the slower accessible process of SO_2_ to the smaller pores in the later time. In this case, the uptake capacity was integrated covering the same exposure time with guaranteed steady state of the later reaction for the same mass of manganese oxide of the same kind. It was found the uptake capacity was dependent on the sample mass and exhibited a linear increase in the range of 0–8.5 mg for α-MnO_2_, 2.0–22.8 mg for β-MnO_2_, 3.0–9.3 mg for γ-MnO_2_, and 1.0 to 15.0 mg for δ-MnO_2_ (Fig. S2). Figure 5 showed the typical uptake curves of SO_2_ on the four crystalline forms of MnO_2_. Once the sample was exposed to SO_2_, a large initial uptake of SO_2_ was observed for δ-MnO_2_, lasting for 150 min until a stable consumption of SO_2_ occurred. The γ-MnO_2_ also showed a substantial uptake of SO_2_, just behind that of δ-MnO_2_. In contrast, the initial uptake of SO_2_ on the other two oxides, α- and β-MnO_2_, was less. The results were consistent with that found by DRIFTS.

**Figure 6:**
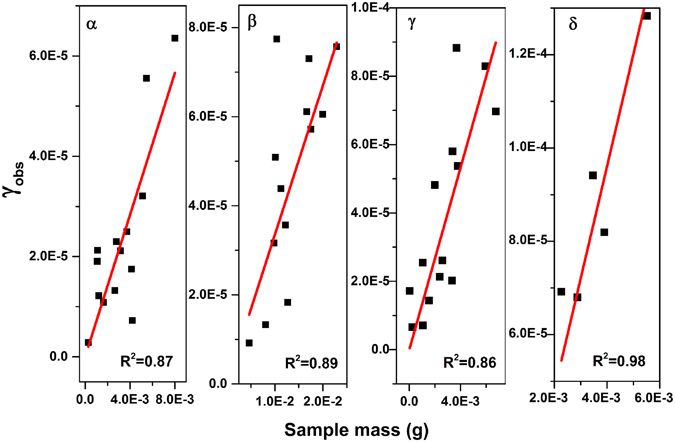
Linear mass dependence for γ_obs_ on manganese oxides under dry conditions.

In the coated-wall flow tube reactor, the uptake coefficient (γ) calculated using the geometric area (γ_obs_) was dependent on the sample mass due to the multilayer thickness generated in the tube. Thus the dependence of γ_obs_ on the sample mass was obtained to determine the probe depth of SO_2_ into the samples as shown in Fig. 6. However, powder samples with porous structures would undergo gas-phase diffusion of reactants into the internal surface of the particles and γ_obs_ represented the upper limit of the uptake coefficient^31^. The γ_obs_ was further corrected with BET surface area according to Equation (2), denoted as γ_c_. Since it was uncertain concerning the valid area available for SO_2_ uptake, the γ_c_ here represented the lower limit of the γ.

Table 1 summarized the BET areas, pore volumes, crystal sizes, SO_2_ uptake capacities and SO_2_ uptake coefficients for the different crystalline manganese oxides. The BET area and pore volume demonstrated a positive correlation with the uptake capacity of SO_2_ per unit of mass due to the large areas and pores in the vicinity of active sites available for the adsorption of SO_2_ and storage of formed sulfate, respectively, but were not the determinant factors given the abnormal phenomenon occurring on α- and β-MnO_2_ once the uptake capacity was normalized to per BET area^47^. If the molecule number of sulfate was considered equal to that of SO_2_ assuming all of the SO_2_ taken up by the manganese oxide converted into sulfate, all of the values for those four manganese oxides, however, were lower than 1.4 × 10^19^ molecules m^−2^ for an ideal monolayer of sulfate, indicating that the catalytic reaction by the redox of MnO_2_ cannot be assured in the present study. In fact, the molecule number of sulfate ion formed on δ-MnO_2_ after saturation with SO_2_ in the DRIFTS experiment calibrated with IC was (1.03 ± 0.10) ×10^18^ molecules m^−2^, obviously lower than (1.63 ± 0.08) ×10^18^ molecules m^−2^ of the uptake amount of SO_2_ measured through flow tube experiments^31^. This difference was possibly due to that flue tube experiments gave a total uptake of SO_2_, including physical and chemical adsorption of SO_2_, while DRIFTS experiment just gave the chemical adsorption of SO_2_. In addition, sulfate ion tended to accumulate on top surface of the sample in DRIFTS cell and the diffusion depth of SO_2_ into the DRIFTS cell was uncertain in this study, therefore, the amount of sulfate obtained by DRIFTS experiments might be underestimated. A diffusion of SO_2_ into inner layers of the samples occurred because the initial uptake coefficients using geometric area (γ_obs_) were found to be dependent on the BET area, with largest γ_obs_ of (2.42 ± 0.13) ×10^−2^ for δ-MnO_2_ and smallest γ_obs_ of (7.07 ± 0.72) ×10^−2^ for β-MnO_2_. After the uptake coefficients were normalized to BET area, δ-MnO_2_ showed the largest corrected uptake coefficient (γ_c_), with (1.48 ± 0.21) ×10^−6^; in contrast, the **γ**_c_ of α-, β- and γ-MnO_2_ was one order of magnitude smaller than that of δ-MnO_2_, i.e., the oxidation reactivity of α-, β- and γ-MnO_2_ was almost the same when the BET area was used as the reactive area. The results indicated that the reactivity of MnO_2_ towards the uptake of SO_2_ was to some extent determined by the chemical properties of the oxides.

**Figure 7:**
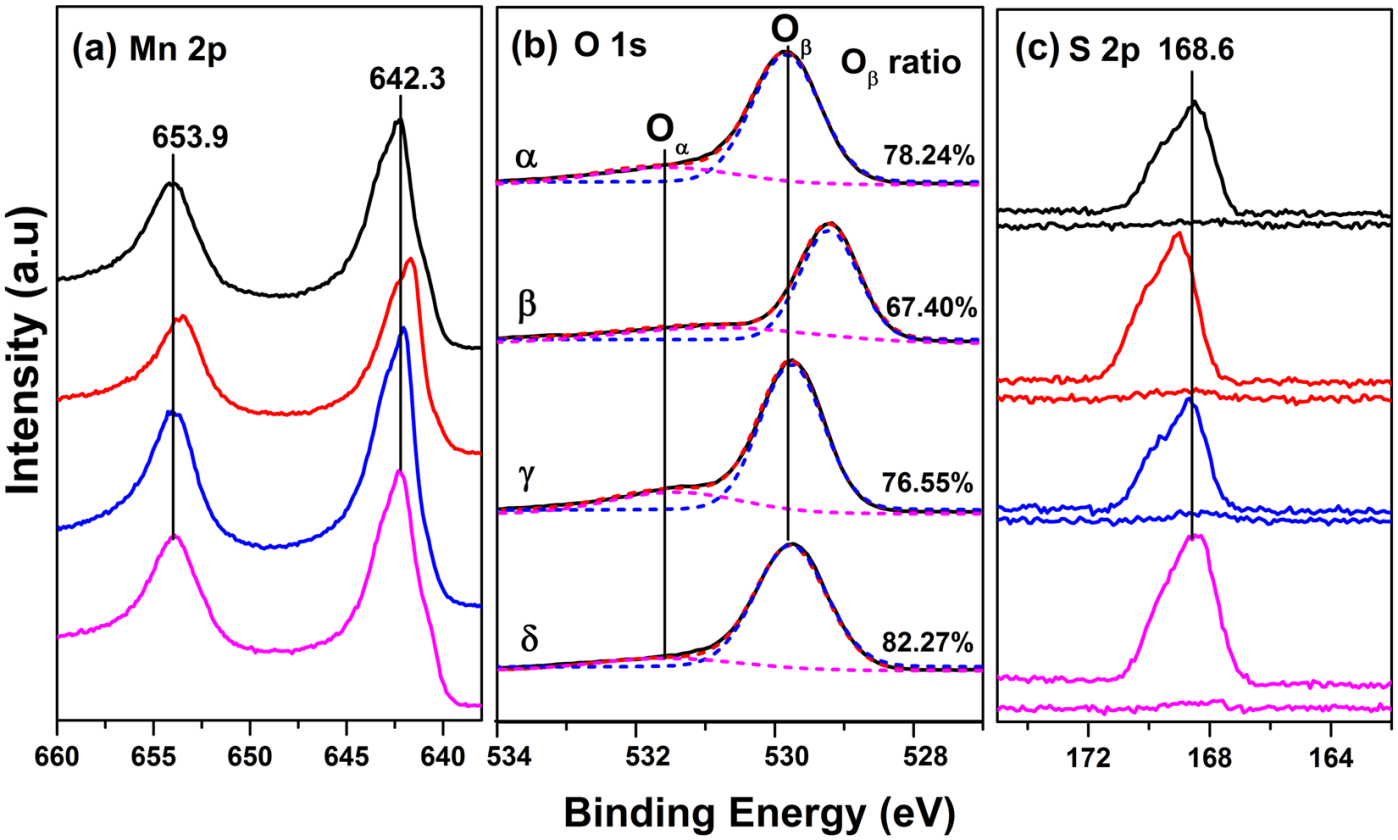
XPS spectra of α-, β-, γ- and δ-MnO_2_: (**a**) Mn 2p, (**b**) O 1 s, (**c**) S 2p.

To explore the influence of surface atomic state on the oxidation activity, XPS spectra were recorded for MnO_2_ with different structures, as shown in Fig. 7. Two characteristic peaks located at 653.9 and 642.3 eV ascribed to Mn 2p_1/2_ and Mn 2p_3/2_ appeared, indicating that Mn^4+^ dominated on the surface^39^. The O 1 s spectrum was deconvoluted into two peaks, with one binding energy at 531.5 eV assigned to surface adsorbed oxygen (denoted as O_α_) and another at 529.7 eV assigned to lattice oxygen (denoted as O_β_)^38, 39, 48^. Noting that adventitious carbon during the probing of X-ray radiation contained C=O and C-O-C groups, as shown in C 1 s spectra (Fig. S3), those oxygen-containing species also contributed to the appearance of O_α_ but had little impact on the ratio of O_α_ to O_β_ due to their almost same small percents occupying the total adventitious carbon (ca. 11%). The relative concentrations of O_β_/(O_α**+**_O_β_) were listed on the right side of Fig. 7(b). Previous studies have found that the lattice oxygen concentration corresponded well with the oxidation activity towards HCHO and CO^38, 39^. In the present study, a lattice oxygen test was conducted on δ-MnO_2_ using DRIFTS (shown in Fig. S4). The formation of sulfate kept almost the same in the absence of oxygen with that in the presence of oxygen and even enhanced on the reduced-MnO_2_ without oxygen due to increased mobility of lattice oxygen atoms. Those results confirmed that the main oxidant was also lattice oxygen in this system. As shown in Fig. 7(b), the lattice oxygen concentrations were 78.24%, 67.40%, 76.55% and 82.27% for α, β, γ and δ-MnO_2_, respectively. Clearly, δ-MnO_2_ presented the largest amount of lattice oxygen, which was in good accordance with the highest oxidation reactivity towards SO_2_.

Figure 7 (c) showed the S 2p spectra for fresh and sulfated MnO_2_. The baseline represented the fresh samples, demonstrating no observable sulfur species. After reaction with SO_2_, an evident S 2p peak at 168.6 eV attributed to ${{\rm{SO}}}_{4}^{2-}$ was observed on all of samples^34^. The intensity of the S 2p peak was most prominent on δ-MnO_2_, indicating the strongest oxidation activity towards SO_2_. It was noted that β-MnO_2_ also presented a high S 2p signal, possibly due to the spill-over of sulfur acid to the surface owing to its limited pore structure. The results indicated that surface lattice oxygen was a determinant factor for the formation of sulfate, and the surface pore structure was responsible for the storage of product formed under dry conditions.

According to the discussion above, SO_2_ most probably adsorbed onto lattice oxygen on MnO_2_ as described below:

R1 $${{\rm{SO}}}_{2}({\rm{g}})+{{\rm{MnO}}}_{2}\mathop{\to }\limits^{{{\rm{k}}}_{1}}{{\rm{MnO}}}_{2}{\boldsymbol{\cdot }}{{\rm{SO}}}_{2}^{\ast }(\mathrm{ads})$$

The S^4+^ in adsorbed-SO_3_^2−^ was subsequently oxidized into S^6+^ by Mn^4+^ on δ-MnO_2_ as reported by Chughtai, *et al*. (i.e., reaction R2)^19^.

R2 $${{\rm{MnO}}}_{2}{\boldsymbol{\cdot }}{{\rm{SO}}}_{2}^{\ast }(\mathrm{ads})\mathop{\to }\limits^{{{\rm{k}}}_{2}}{\rm{MnO}}{\boldsymbol{\cdot }}{{\rm{SO}}}_{3}(\mathrm{ads})$$

DRIFTS spectra showed that a small amount of water still remained on the surface (Fig. S5). Therefore, SO_3_ attached to the Mn atom would transform into H_2_SO_4_ once connecting to water molecules (shown in Equation R3).

R3 $${\rm{MnO}}{\boldsymbol{\cdot }}{{\rm{SO}}}_{3}(\mathrm{ads})+{{\rm{H}}}_{2}{\rm{O}}\mathop{\to }\limits^{{{\rm{k}}}_{3}}{\rm{MnO}}+{{\rm{H}}}_{2}{{\rm{SO}}}_{4}$$

Steady reaction of SO_2_ was observed in the flow tube reactor and increasing formation of SO_4_^2−^ was found in the DRIFTS investigation, indicating that the Mn^2+^ in Equation (R3) might be regenerated into Mn^4+^ after oxidation of SO_2_. XPS results revealed that activated lattice oxygen was a main oxidant in this system, and MnO_2_ can be recovered by reaction between MnO and lattice oxygen. In addition, gaseous oxygen can adsorb on oxygen vacancy sites to dissociate into adsorbed oxygen atoms to provide activated lattice oxygen (as shown in Equation R4)^29^.

R4 $${{\rm{O}}}_{2}+{\rm{O}}\,({\rm{vacancy}})\to 2[{\rm{O}}]$$

Once the surface was saturated with H_2_SO_4_, the localized MnO would transform into MnSO_4_ and hence limited the buildup of SO_4_^2−^ further,

R5 $${\rm{MnO}}+{{\rm{H}}}_{2}{{\rm{SO}}}_{4}\mathop{\to }\limits^{{{\rm{k}}}_{4}}{{\rm{MnSO}}}_{4}(\mathrm{ads})+{{\rm{H}}}_{2}{\rm{O}}$$

According to the discussion above, the formation rate of sulfate can be described by a general equation:

1 $${\rm{r}}=\frac{{d[\mathrm{SO}}_{4}^{{\rm{2}}-}]}{{\rm{dt}}}=-\frac{d[{{\rm{H}}}_{2}{{\rm{SO}}}_{4}]}{{\rm{dt}}}=\frac{d[{\rm{MnO}}{\boldsymbol{\cdot }}{{\rm{SO}}}_{3}]}{{\rm{dt}}}={{\rm{k}}}_{2}[{{\rm{MnO}}}_{2}{\boldsymbol{\cdot }}{{\rm{SO}}}_{2}^{\ast }]$$

No sulfite species was observed in the DRIFTS spectra, suggesting that the adsorbed SO_2_ was quickly oxidized into sulfate, i.e., the net formation rate of sulfite equaled zero:

2 $$\frac{{d[\mathrm{MnO}}_{2}{\boldsymbol{\cdot }}{{\rm{SO}}}_{2}^{\ast }]}{{\rm{dt}}}={{\rm{k}}}_{1}[{{\rm{SO}}}_{2}][{{\rm{MnO}}}_{2}]-{{\rm{k}}}_{2}{[\mathrm{MnO}}_{2}{\boldsymbol{\cdot }}{{\rm{SO}}}_{2}^{\ast }]={\rm{0}}$$

Thus:

3 $$r=\frac{{d[H}_{2}{{\rm{SO}}}_{4}]}{{\rm{dt}}}={{\rm{k}}}_{1}[{{\rm{SO}}}_{2}][{{\rm{MnO}}}_{2}]$$

Equation (3) showed that the reaction was first order with respect to SO_2_. To clarify the reaction order of SO_2_ on manganese oxides, for instance, on δ-MnO_2_, the sulfate absorbance bands in DRIFTS experiments were calibrated with ion chromatography (Fig. S6). Noting that the intensity of the sulfate absorbance bands ranging 1552–782 cm^−1^ on δ-MnO_2_ (Fig. 3) in the growth stage was proportional to the sulfate concentration, the initial formation rate can be translated from the integrated area to sulfate ions per unit time by a conversion factor *f*. The conversion factors for dry and wet conditions (RH = 40%) were different, as shown in Fig. S6(a). In our study, *f* was calculated to be 8.83 × 10^18^ (ions g^−1^ integrated absorbance units^−1^) for δ-MnO_2_ compromising those conversion factors for dry and wet conditions so as to be applied for different RHs ranging from 0 to 65% (Fig. S6(b)). In a future study, the relationship of the conversion factor and the specific RH should be verified. The reaction order of SO_2_ was hence obtained from the slope of the bilogarithmic curve of sulfate formation rate vesus SO_2_ concentration. As shown in Fig. S7, the reaction order of SO_2_ was determined as 1.20 ± 0.07, consistent with the result of Equation (3).

The active sites, i.e., lattice oxygen on the sample, reacted with SO_2_ to form SO_3_, which would combine with surface-adsorbed water quickly and then migrated into the nearby pores as sulfuric acid^47^. Therefore, the formation rate of sulfuric acid was relatively fast at the early stage due to the large amount of active sites and pores available for SO_2_. Once the pores were saturated with sulfuric acid, the active sites would be poisoned to form MnSO_4_, decelerating the reaction rate. The variation in SO_2_ uptake capacity per unit of mass for different crystalline forms of MnO_2_ in the order of δ- > γ- > α- **≈** β-MnO_2_ was basically in accord with their lattice oxygen concentrations and pore volumes.

In addition, the difference in crystal structure (shown in Fig. S1 and Fig. 2) may also be one of the reasons for the different activity. The correlation between the activity and the phase structure of MnO_2_ has been discussed in detail^38–40^. Liang *et al*. observed that δ-MnO_2_, with a 2D layer built up by sheets of edge-sharing MnO_6_ octahedra, favored the adsorption of CO^39^. In contrast, β-MnO_2_, with narrow (1 × 1) channels, cannot accommodate reactants^39, 40^. Our results were consistent with those reported previously, in that δ-MnO_2_ performed best while β-MnO_2_ performed worst with regards toward SO_2_ adsorption per unit of mass. The α-MnO_2_ structure with 1D (2 × 2) and (1 × 1) channels, consisting of double chains of edge-sharing MnO_6_ octahedra, was generally reported to be more active than γ-MnO_2_, which was a random intergrowth of ramsdellite (1 × 2) and pyrolusite (1 × 1) channels^38–40^. However, we had the reverse results in this work. The reason for this discrepancy remained unknown, and was possibly due to differences in reactants and reaction conditions.

### Reactions under wet conditions

**Figure 8:**
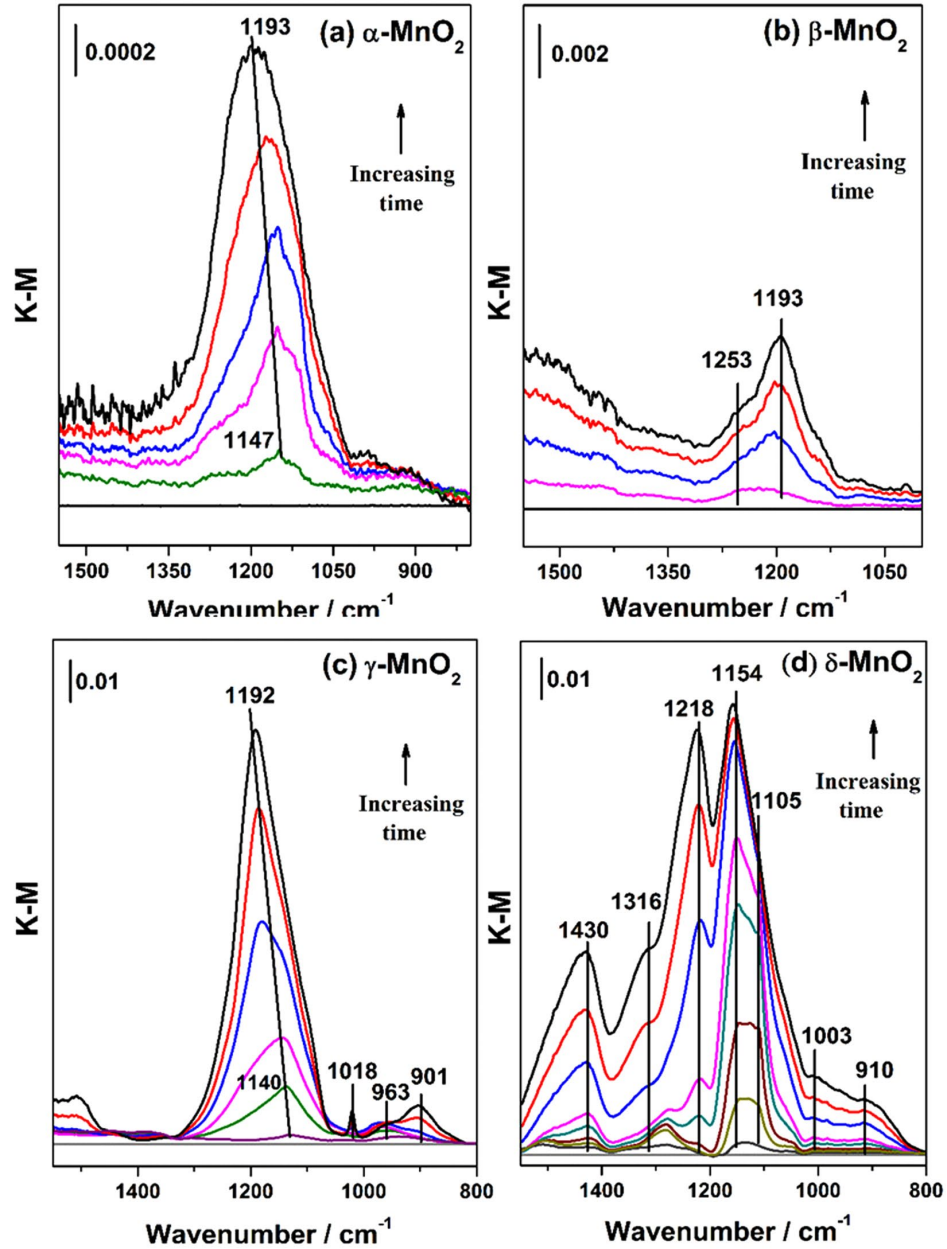
DRIFTS spectra recorded on manganese oxides exposed to 40 ppmv SO_2_ as a function of time under 40% RH. The total flow was 100 mL/min and reaction time was 60 min.

Water plays an important role in the heterogeneous atmospheric reactions^16^. To explore the effect of water, DRIFTS spectra for α-, β-, γ- and δ-MnO_2_ exposed to SO_2_ under wet conditions were recorded as a function of time, as shown in Fig. 8. It was evident that the chemical state of sulfate species changed compared to that under dry conditions due to the influence of water. Both α- and γ-MnO_2_ showed only one band above 1100 cm^−1^, indicating that a monodentate sulfate structure formed on the surfaces^41^. In addition, the band at around 1140 cm^−1^ blue-shifted to 1192 cm^−1^, suggesting that accumulation of sulfate species occurred with increasing time by the promoting effect of surface-adsorbed water^45^.

For β-MnO_2_, a bidentate sulfato-surface complex formed in the presence of water, as two bands at 1253 and 1193 cm^−1^ appeared. In the case of δ-MnO_2_, in addition to the formation of bidentate sulfate at bands of 1218, 1154, 1003 and 910 cm^−1^, a new band at 1105 cm^−1^, assigned to the free sulfate ions, grew in intensity. The results suggested that an aqueous film may form on the surface. In addition, the increased amount of polymeric or accumulated-sulfate species represented by bands of 1430 and 1316 cm^−1^ implied that water accelerated the formation of sulfate.

**Figure 9:**
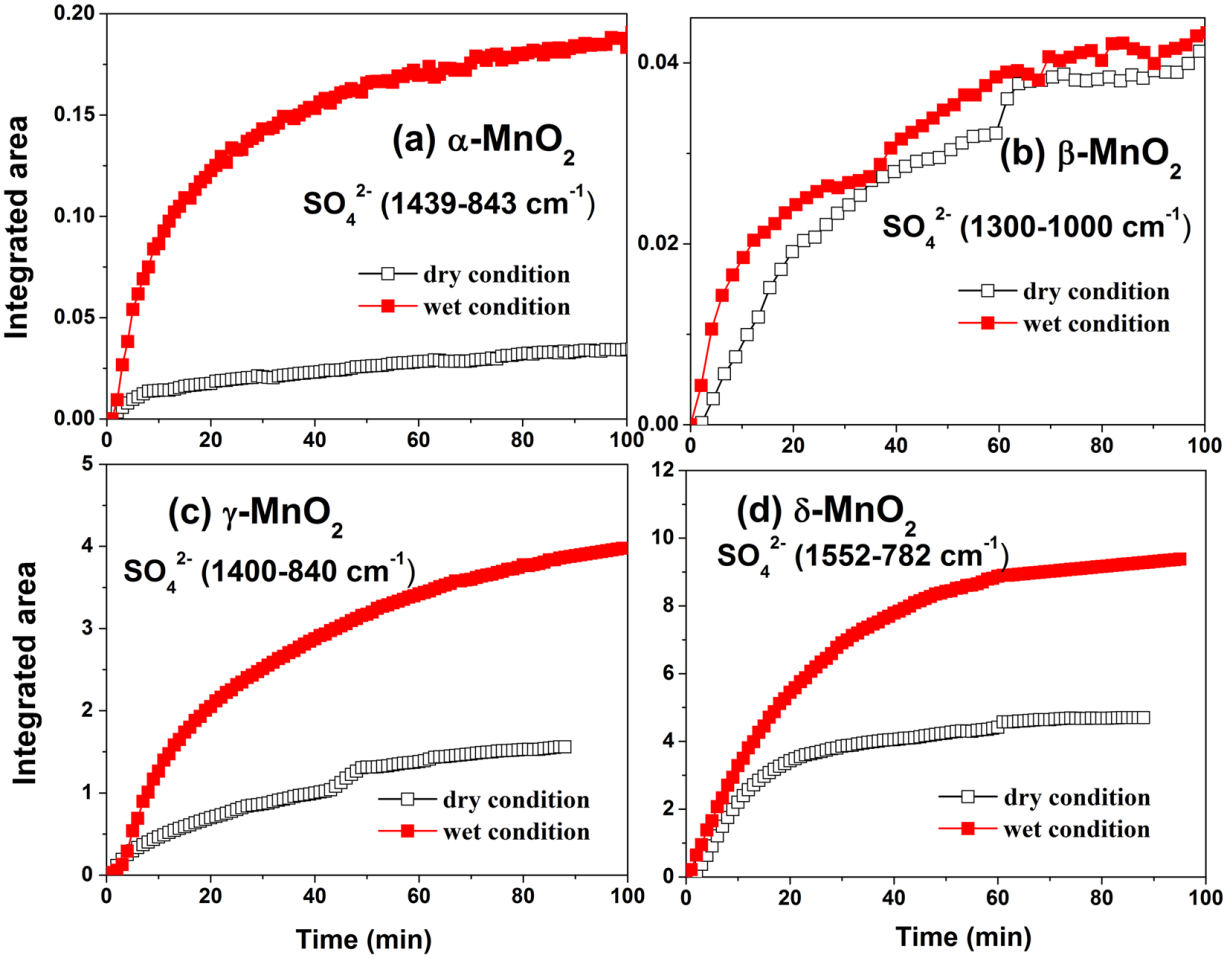
Comparison of integrated areas for the sulfate species formed on different crystal manganese oxides between dry and 40% RH conditions. α-MnO_2_ (1439–843 cm^−1^), β-MnO_2_ (1317–1112 cm^−1^), γ-MnO_2_ (1400–840 cm^−1^) and δ-MnO_2_ (1552–782 cm^−1^).

As shown in Fig. 9, the integrated absorbance areas representing the sulfate amounts formed under dry (<1%) and wet conditions (40% RH) were compared for each crystalline manganese oxide. Clearly, the presence of water led to a higher amount of sulfate on the samples except for β-MnO_2_ due to its poor signal in the sulfate absorption region. At the initial stage, the sulfate concentration grew linearly with time and became more rapidly under wet condition than under dry condition, i.e., water improved the initial rate of sulfate formation. Since a large amount of active sites were available at the beginning of the reaction, which can be seen as a constant, the rate of sulfate formation only depended on the concentrations of SO_2_ and H_2_O. When the active sites were covered with sulfate species, the reaction rate would be influenced by the products. Therefore, to elucidate the reaction mechanism under wet condition, further investigation concerning the initial rate of sulfate formation as a function of RH ranging from 6% to 65% was conducted on δ-MnO_2_.

**Figure 10:**
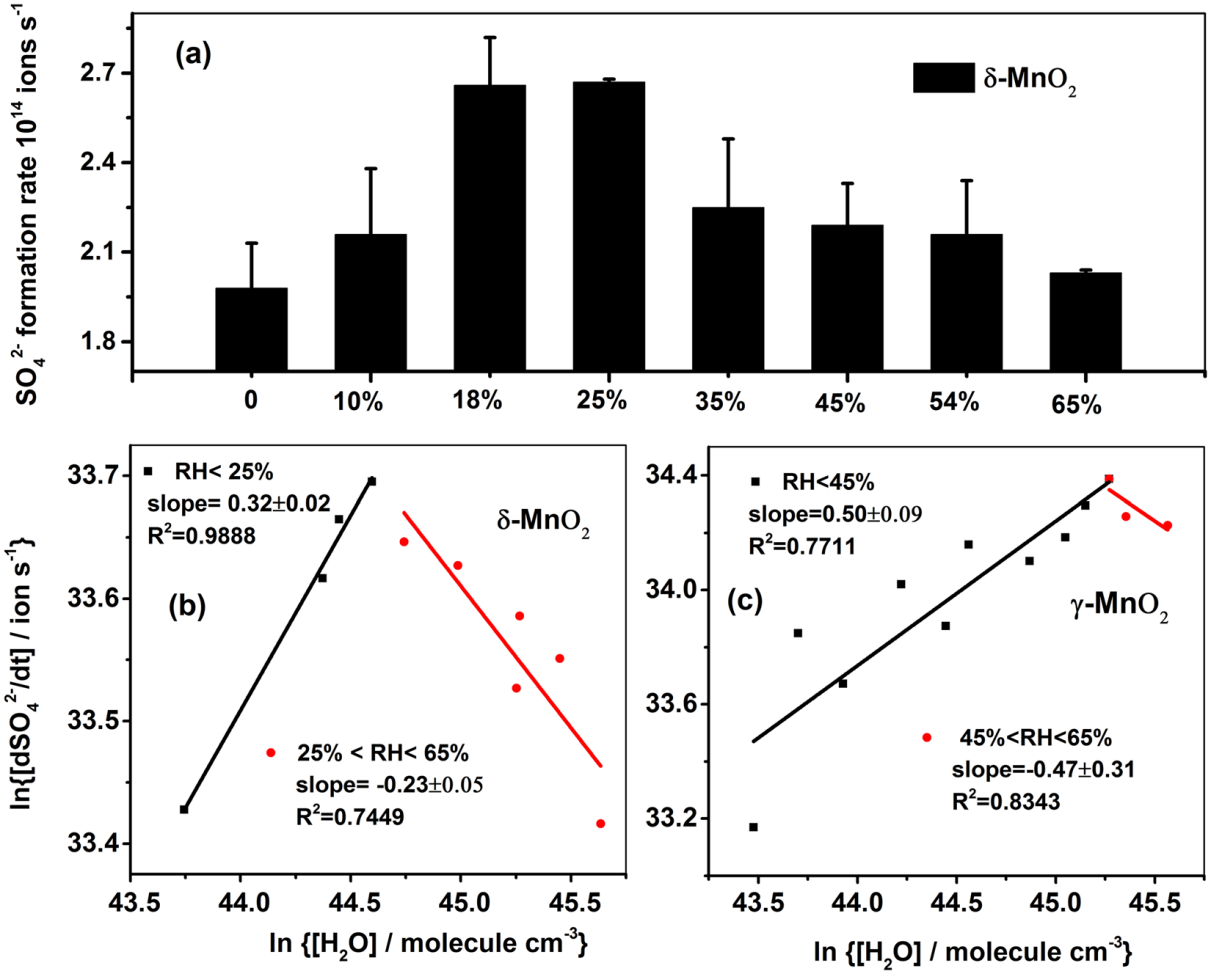
(**a**) Sulfate formation rate on δ-MnO_2_ at different RHs, and bilogarithmic plots of the sulfate formation rate versus [H_2_O] on (**b**) δ-MnO_2_ and (**c**) γ-MnO_2_.

As can be seen in Fig. 10(a), the initial formation rate of sulfate on δ-MnO_2_ first increased with RH and then decreased when the RH was above 25%. Figure 10(b) gave the reaction order of H_2_O from a bilogarithmic plot slope of the SO_4_^2−^ formation rate versus the H_2_O concentration at a constant SO_2_ concentration. The reaction order of H_2_O (g) was 0.32 with RH < 25% and −0.23 with RH from 25% to 65%. Similar phenomenon was also observed on γ-MnO_2_ though the maximum value for the sulfate formation rate was reached at RH = 45%, as shown in Fig. 10(c). The reaction order of H_2_O on γ-MnO_2_ was 0.50 at RH < 45% and −0.47 at RH = 45–65%. At low RH, the positive reaction orders with respect to H_2_O and SO_2_ indicated that SO_2_ oxidation on MnO_2_ proceeded through Langmuir-Hinshelwood mechanism, where dissolved SO_2_ in limited water layers dissociated as follows^22, 36, 49^,

R6 $${{\rm{SO}}}_{2}({\rm{ads}})+{{\rm{nH}}}_{2}{\rm{O}}({\rm{ads}})\mathop{\to }\limits^{{{\rm{k}}}_{5}}{{\rm{2H}}}^{+}+{{\rm{SO}}}_{3}^{{\rm{2}}-}(\mathrm{aq})+(n-1){{\rm{H}}}_{2}{\rm{O}}({\rm{ads}})$$

Previous study demonstrated that Mn^4+^ on MnO_2_ was always first reduced to a lower oxidation state of Mn^2+^ on MnO in the localized reaction as well as in the catalytic reaction of SO_2_^19^. XPS spectra in the present study showed that the Mn 2p bands shifted towards lower binding energies (ca. 0.3 eV) after reaction with SO_2_ in the presence of water, indicating that Mn^4+^ acted as an oxidant during this process (Fig. S8). Therefore,

R7 $${{\rm{MnO}}}_{2}+{{\rm{SO}}}_{3}^{{\rm{2}}-}(\mathrm{aq})\mathop{\to }\limits^{{{\rm{k}}}_{6}}{\rm{MnO}}+{{\rm{SO}}}_{4}^{{\rm{2}}-}(\mathrm{aq})$$

Since the adsorption of SO_2_ was the rate-limiting step and sulfite was the intermediate product, the formation rate of sulfate under wet conditions could be expressed as the following,

4 $${\rm{r}}=\frac{{d[\mathrm{SO}}_{4}^{{\rm{2}}-}]}{{\rm{dt}}}={{\rm{k}}}_{6}[{{\rm{MnO}}}_{2}{][\mathrm{SO}}_{3}^{{\rm{2}}-}]={{\rm{k}}}_{5}[{{\rm{SO}}}_{2}]{[{{\rm{H}}}_{2}{\rm{O}}]}^{n}$$

Equation (4) showed that the reaction order of SO_2_ was pseudo-first-order, in line with the experimental results (Fig. S7).

At low RH, water was favorable for the sulfate formation. However, once the RH was further increased, excessive water may cover the active sites and prevent the recovery of Mn^4+^ from Mn^2+^ by lattice oxygen or gaseous oxygen, thus decreasing the initial reaction rate. It was noticed that inhibition effect of water on the formation rate of sulfate started at a lower RH on δ-MnO_2_ than on γ-MnO_2_, which was possibly due to the different crystal structures. For δ-MnO_2_, a 2D layered structure with larger dimension of channels embedded a larger amount of H_2_O onto the surface of the sample^39^. In contrast, γ-MnO_2_ with irregular channels was narrow for the entrance of H_2_O. Therefore, under wet conditions, the H_2_O concentration was possibly easier to adsorb on δ-MnO_2_ than on γ-MnO_2_ and hence lowered the formation rate of sulfate for the former at lower RH.

## Conclusion

The heterogeneous reaction of SO_2_ on MnO_2_ with different crystal structures was investigated under dry and wet conditions. Under dry conditions, DRIFTS spectra showed that the chemical state of sulfate species varied for different crystalline forms of MnO_2_, where accumulation of sulfate occurred more clearly on γ- and δ-MnO_2_ than on α- and β- MnO_2_. It was found that the reactivity of MnO_2_ towards SO_2_ adsorption decreased in the order of δ-** > **γ-** > **α- **≈** β-MnO_2_ by using a flow tube reactor and DRIFTS. Under wet conditions, adsorbed water changed the chemical form of sulfate as well as accelerating the formation rate of sulfate. On δ-MnO_2_, surface-adsorbed water increased the initial rate of sulfate formation at low RH (**≤**25%), whereas it lowered the formation rate of sulfate species when the RH was further increased. Similar phenomenon was also found on γ-MnO_2_, with a maximum value at 45% RH.

In regions with anthropogenic impacts, airbone dust emitted from polluted soil or water sources may bear highest mass ratio of Mn, assuming a largest fraction of 0.13% in the mineral dust^37, 50^. Here, taking the γ_c_ of δ-MnO_2_, i.e., (1.48 ± 0.21) ×10^−6^, as the largest uptake of SO_2_ on all those true manganese oxides, the possible formation rate of sulfate would be lower than 1.94 × 10^6^ molecules cm^−3^ per day, given a SO_2_ concentration of 10 ppbv and the surface area of mineral dust to be 6.3 × 10^−6^ cm^2^ cm^−3^ in polluted areas and seasons^31^. This value can be negligible in the atmosphere and implied that the heterogeneous reaction of SO_2_ on manganese oxides is not an important process.

Nevertheless, the assessment of SO_2_ oxidation in this study should be an lower limit for the atmospheric relavance. A recent study by Li *et al*. showed that transition metals in heterogeneous catalytic oxidation of SO_2_ in aerosol water could play an important role in the formation of sulfate during haze days in China^11^. However, the largest uptake coefficient of SO_2_ on manganese oxides measured in the present study was one order of magnitude lower than that for Fe^3+^-catalyzed SO_2_ oxidation in aerosol water assumed by Li *et al*.^11^. This is because the Mn metal was in the bulk phase in the present study while Fe was considered as Fe^3+^ ion in aqueous phase at high RH in Li *et al*.’s study. Since manganese oxides or manganese-containing aerosols can release Mn^2+^ ion in aerosol water to catalyze SO_2_ oxidation in aqueous phase and accelerated the formation of sulfate^19^, the contribution of manganese oxides to sulfate formation might be underestimated missing the role of Mn^2+^ ion in catalytic oxidation of SO_2_ in this study. In addition, the oxidation of SO_2_ by manganese oxides in this study was auto-inhibited and the surface was deactivated with time due to increased acidity. However, alkaline gases like NH_3_ in the atmopshere may maintain the reaction rate and enhanced the formation of sulfate since enough alkalinity was assumed to significantly promote aqueous oxidation of SO_2_^9, 11, 51^. Besides, aging of particulate matter due to exposure to high concentrations of gaseous pollutants in heavily polluted regions occurred very rapidly, thus enabling thick coating of other aerosol constituents, mainly organic species, onto the surface of PM within a very short time as reported by Peng *et al*.^52^. The coating of hygroscopic components would enhance the surface hygroscopicity and possibly promoting aqueous oxidation of SO_2_ further on manganese oxides^9, 53^. Literature reports showed that the uptake coefficient of SO_2_ increased significantly by the presence of water and reached an upper limit of 10^−2^–10^−1^ in aqueous phase, which was much higher than that reported by Li *et al*. and our result^4, 11, 54^. Therefore, the Mn^2+^ ion, alkaline gases (such as NH_3_) and other aerosol constituents need to be highlightened in future research to comprehensively understand the heterogeneous oxidation of SO_2_ on Mn-containing aeresols at elevated RHs.

Results from this study suggest that the morphology of the mineral dust and the relative humidity outsides may play significant roles in the transformation of SO_2_. An early study found that macroscopic properties like bulk denstiy, specific gravity and area of manganese oxides had an important impact on the adsorption of SO_2_ at 300 °C and introduction of 3.4% volume moisture would contribute to this process^55^. In this study, it was further found that microscopic structure like crystalline phase and morphology also exerted an influence on the oxidation of SO_2_ at ambient temperature. More importantly, relative humidty would not promote the oxidation of SO_2_ through the whole range and it might inhibite the conversion of SO_2_ at high RH though with a slightly higher initial formation rate of sulfate than that under dry condition. Therefore, an establishment of the relationship between the morphology, RH and the activity towards the uptake of SO_2_ should be included for different type of mineral dust in future model simulations.

## Methods

### Materials

Manganese dioxides with four crystal structures, α, β, γ and δ, used in this study were prepared by a hydrothermal method according to a procedure reported in our previous study^38^. X-ray diffraction (XRD) equipped with Cu Kα (λ = 0.15406 nm) radiation source was applied to analyze the bulk crystalline phase of MnO_2_ using a computerized PANalytical X’Pert Pro diffractometer system. High Resolution-Transmission electron microscopy (HR-TEM) was performed on a FEI Tecnai G^2^ F20 electron microscope operating at 200 kV with supplied software for automated electron tomography. The samples were dispersed in ethyl alcohol and sonicated for 30 min, and then transferred to carbon-coated copper grids. Excess solution was evaporated at room temperature. Brunauer-Emmet-Teller (BET) adsorption isotherm measurements were carried out using a Quantachrome Quadrasorb SI-MP system. The BET areas and average particle sizes for the four manganese oxides are listed in Table 1.

### *In situ* DRIFTS

The Infrared spectrum of the particle surfaces was collected using *in situ* DRIFTS (Nicolet is50, Thermofisher Scientific Co., USA) during reactions. The samples were placed into a ceramic sample holder in the chamber. All the samples were pretreated at 473 K for 60 min to remove adsorbed species in a 100 mL min^−1^ flow of synthetic air (80% N_2_ and 20% O_2_), and then the temperature was cooled down and maintained at 303 K using a temperature controller. When the background spectrum of the fresh sample reached steady state, a mixture of 40 ppmv SO_2_ and synthetic air was introduced into the chamber at a flow of 100 mL min^−1^, during which the IR spectra were recorded at a resolution of 4 cm^−1^ for 30 scans in the spectral range of 4000 to 600 cm^−1^. For reactions under wet conditions, the relative humidity was regulated by adjusting the mix ratio of dry nitrogen to nitrogen bubbled through pure water. The humidity value was monitored using a hygrometer (CENTER 314). All of the measurements were repeated at least three times.

### Flow Tube Reactor

The uptake experiments were performed in a 20 cm × 1.0 cm (i.d.) horizontal cylindrical coated-wall flow tube reactor, which has been described in detail elsewhere^56, 57^. The temperature was maintained at 298 K by circulating water through the outer jacket of the flow tube reactor. Synthetic air as the carrier gas was introduced in the flow tube reactor at 770 ml min^−1^ to ensure a laminar regime at ambient pressure. SO_2_ was introduced into the gas flow through a movable injector with 0.3 cm radius. The SO_2_ concentration was kept at 205 ± 5 ppb, measured by a SO_2_ analyzer. Before experiments, the powder samples were suspended in ethanol and dripped uniformly into the Pyrex flow tube, and then dried overnight in oven at 373 K. No uptake of SO_2_ was observed when the reactant gases were introduced into the blank quartz tube.

The reaction kinetics (*k*_obs_) of SO_2_ can be described in terms of the uptake coefficient, assuming a pseudo first-order reaction with respect to the concentration of SO_2_ according to Equation (5):

5 $${k}_{{\rm{obs}}}=\frac{{{\rm{\gamma }}}_{{\rm{obs}}} < {\rm{c}} > }{2{{\rm{r}}}_{{\rm{tube}}}}$$

where γ_obs_, <c> and r_tube_ refer to geometric uptake coefficient, average molecular velocity of SO_2_ and the flow tube radius. The geometric inner surface area of the whole sample was used to calculate the γ_obs_ because the injector was pulled back to the end of the sample tube. The gas phase diffusion limitation was corrected using the Cooney-Kim-Davis (CKD) method^58^. There exists a probability of diffusion of SO_2_ into underlying layers of the sample, thus the corrected uptake coefficient (γ_c_) normalized to the BET surface area in a linear increase regime of γ_obs_ *vs* the sample mass was obtained as follows:

6 $${{\rm{\gamma }}}_{{\rm{c}}}=\frac{{{\rm{\gamma }}}_{{\rm{obs}}}\times {{\rm{S}}}_{{\rm{geom}}}}{{{\rm{S}}}_{{\rm{BET}}}\times {\rm{M}}}$$

where S_geom_ is the geometric area of the flow tube reactor, S_BET_ is the BET surface area of the sample and M is the sample mass.

### Ion chromatography (IC)

The products formed on the particles after reaction with SO_2_ in the *in situ* chamber cell were analyzed by means of ion chromatography. The reacted sample particles were extracted by sonication with 10 mL ultrapure water (specific resistance ≥18.2 MΩ cm) for 30 min. The leaching solution was filtered with a 0.22 μm PTFE membrane and then analyzed using a Wayee IC-6200 ion chromatograph equipped with a TSKgel Super IC-CR cationic or SI-524E anionic analytical column. An eluent of 3.5 mM Na_2_CO_3_ was used at a flow rate of 0.8 mL·min^−1^.

### XPS

X-ray photoelectron spectroscopy (XPS) profiles were obtained with an AXIS Ultra system (Kratos Analytical Ltd), equipped with Al Ka radiation (1486.7 eV). The C 1 s peak at 284.6 eV was used as an internal standard for calibration of binding energies.

## Electronic supplementary material

Supplementary Information
