# Supplementary material for: Heterogeneous Reaction of SO2 on Manganese Oxides: the Effect of Crystal Structure and Relative Humidity
Source: Sci Rep. 2017 Jul 3;7:4550. doi: 10.1038/s41598-017-04551-6 (PMC5495761; doi:10.1038/s41598-017-04551-6)
Supplement: Supplementary file 1 — Supplementary Information [file 41598_2017_4551_MOESM1_ESM.pdf]

Supporting Information for

Heterogeneous Reaction of SO_2 on Manganese Oxides: the Effect of Crystal Structure and Relative Humidity

Weiwei Yang [a, b], Jianghao Zhang †, Qingxin Ma [a, b, *], Yan Zhao [a, b], Yongchun Liu [a, b, c], Hong He [a, b, c, *]

[a] State Key Joint Laboratory of Environment Simulation and Pollution Control, Research Center for Eco-Environmental Sciences, Chinese Academy of Sciences, Beijing 100085, China

[b] College of Resources and Environment, University of Chinese Academy of Sciences, Beijing 100049, China

[c] Center for Excellence in Urban Atmospheric Environment, Institute of Urban Environment, Chinese Academy of Sciences, Xiamen 361021, China

* Corresponding authors

* Fax: 86-10-62849123; tel: 86-10-62849123;

* E-mail: qxma@rcees.ac.cn (Q. Ma); honghe@rcees.ac.cn (H. He)

† present address: Washington State University, 1505 Stadium Way, Pullman, Washington State, 99164, U.S.A.

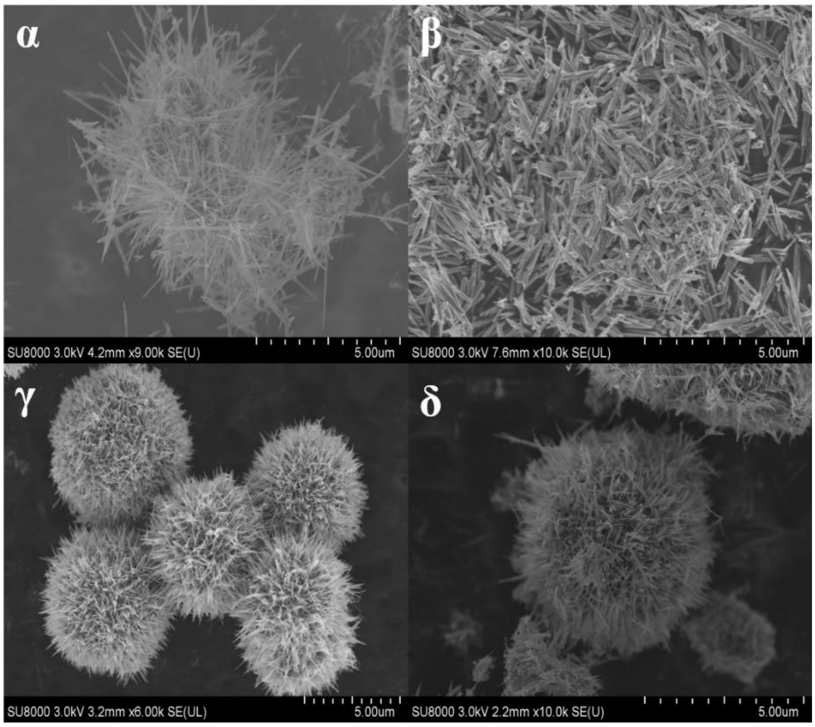

Figure S1. SEM image of α-, β-, γ- and δ-MnO_2

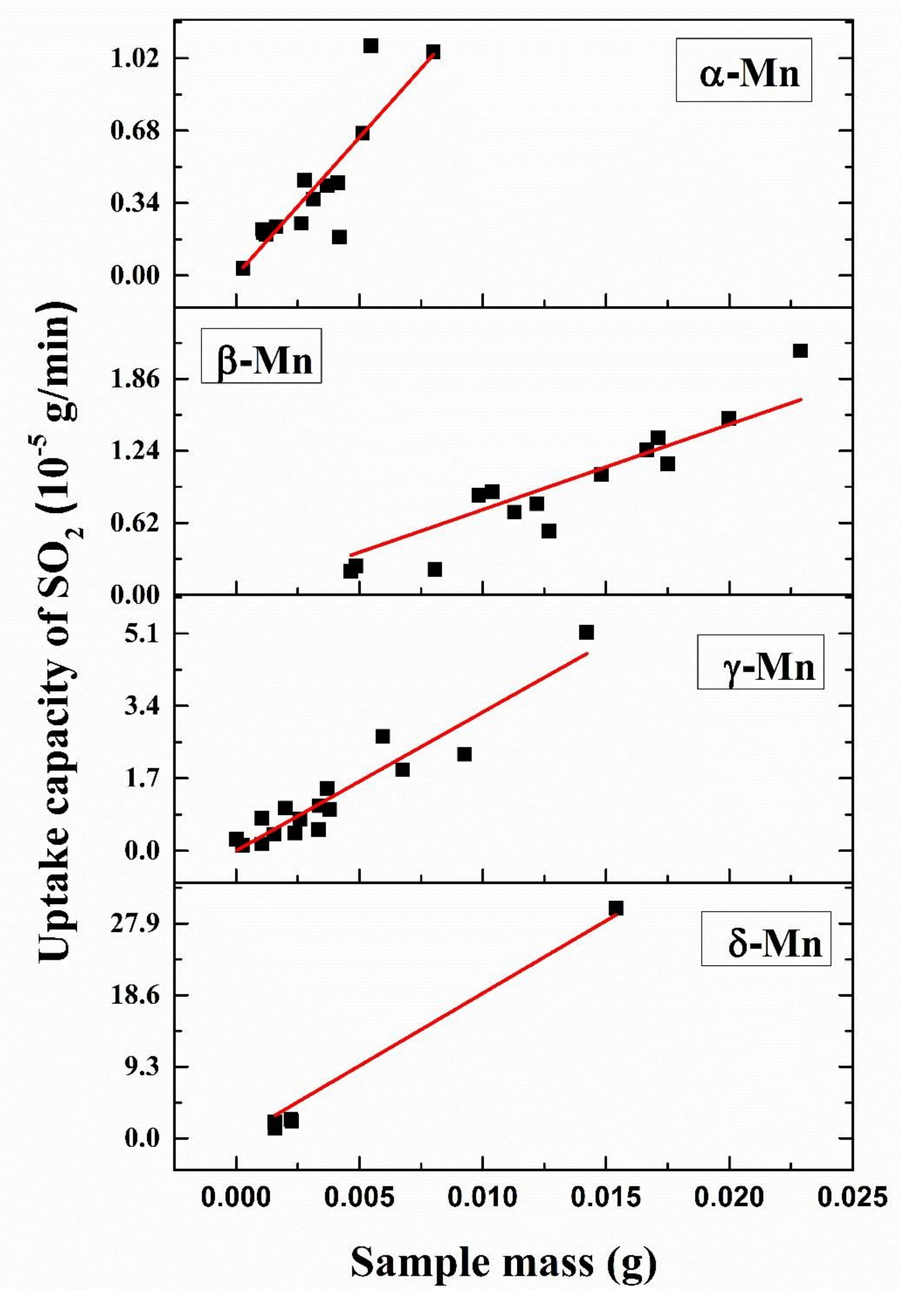

Figure S2. Linear mass dependence for uptake capacity of SO_2 on manganese oxides

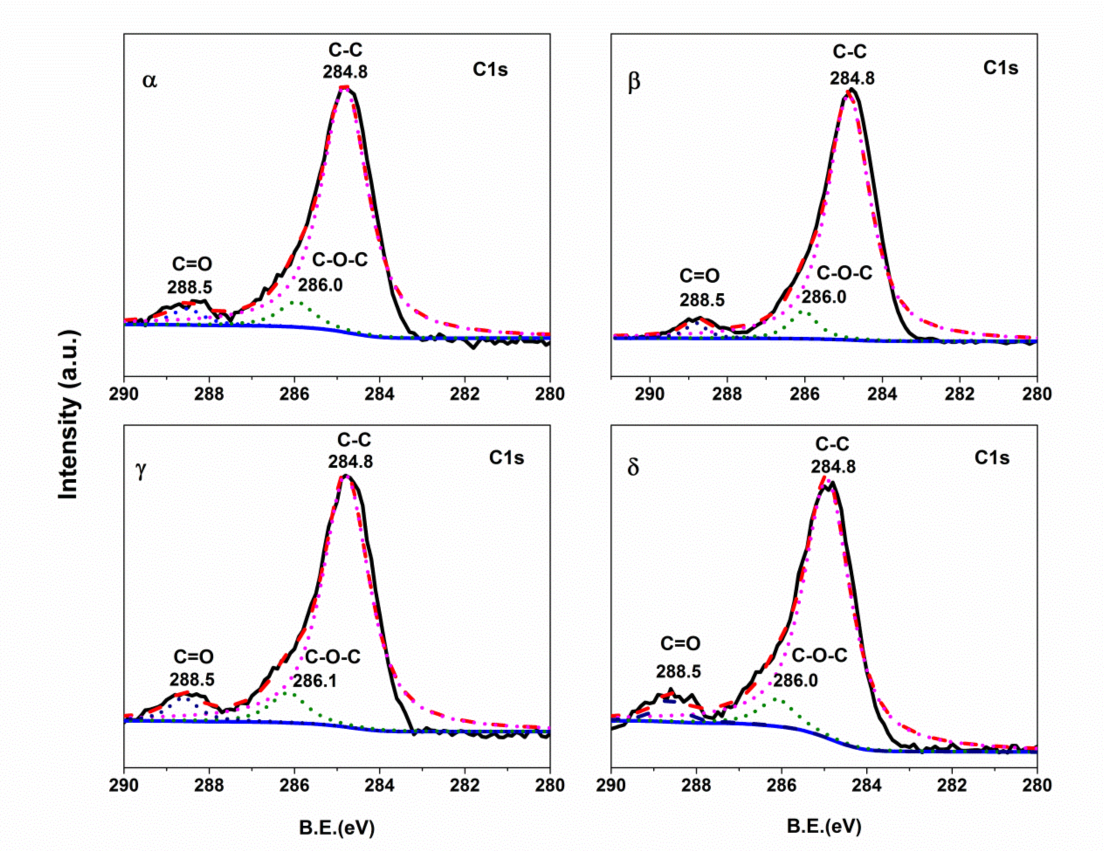

Figure S3. XPS spectra of C1s over manganese oxides

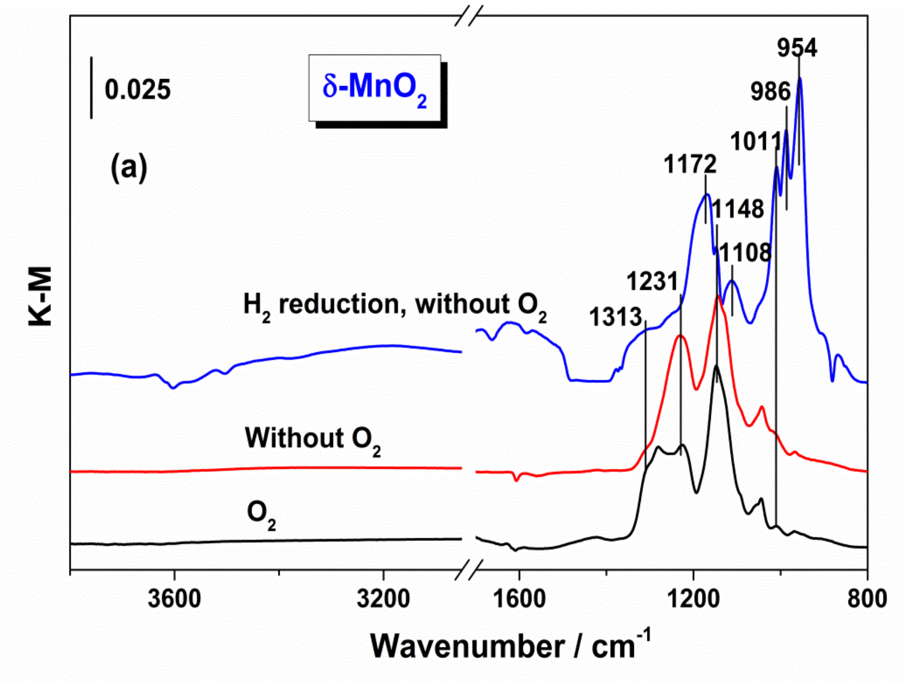

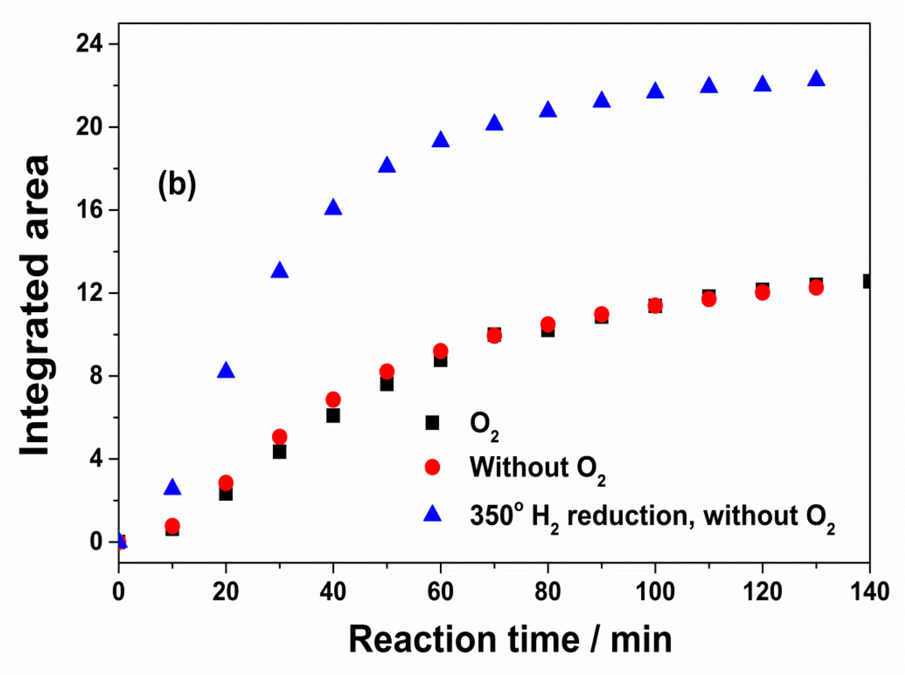

Figure S4. (a) Comparison of DRIFTS spectra for the lattice oxygen test on δ-MnO_2 for the same time under different conditions; (b) Comparison of integrated area for sulfate species formed on the surface of δ-MnO_2

The reaction of δ-MnO_2 with SO_2 was conducted under different conditions. It was found that the adsorption spectra varies little in the absence of gaseous oxygen compared to that in the presence of gaseous oxygen and the integrated areas under those two conditions were almost the same, indicating that gaseous oxygen play a minor role in the oxidation of SO_2. XPS spectra (Fig. 7) show that the surface adsorbed oxygen was considerably low. The H_2-TPR experiment in our previous study confirmed that the main oxygen species on the surface of MnO_2 was lattice oxygen [1]. Therefore, in the present study, lattice oxygen seems the main oxidant in the oxidation of SO_2. After reduction by H_2 at 350 ^{o}C, surface oxygen on the sample was consumed completely while lattice oxygen remained. The sample was then exposed to SO_2 in the absence of oxygen. It was found that the adsorption of SO_2 seems

enhanced in this case because more sulfate species in the low frequency (1011-954 cm^{-1}) region was observed[2]. Fig. S4(b) shows that the integrated area for the sulfate species formed on the H_2 reduced-sample is higher than those of the samples without H_2 reduction indicating a higher reactivity of the H_2 reduced-sample towards the oxidation of SO_2. It was possibly due to the excessive defects by H_2 reduction that enhanced the mobility of the lattice oxygen, thus increasing the oxidation activity. The results above implied that the main oxidant in this system was lattice oxygen.

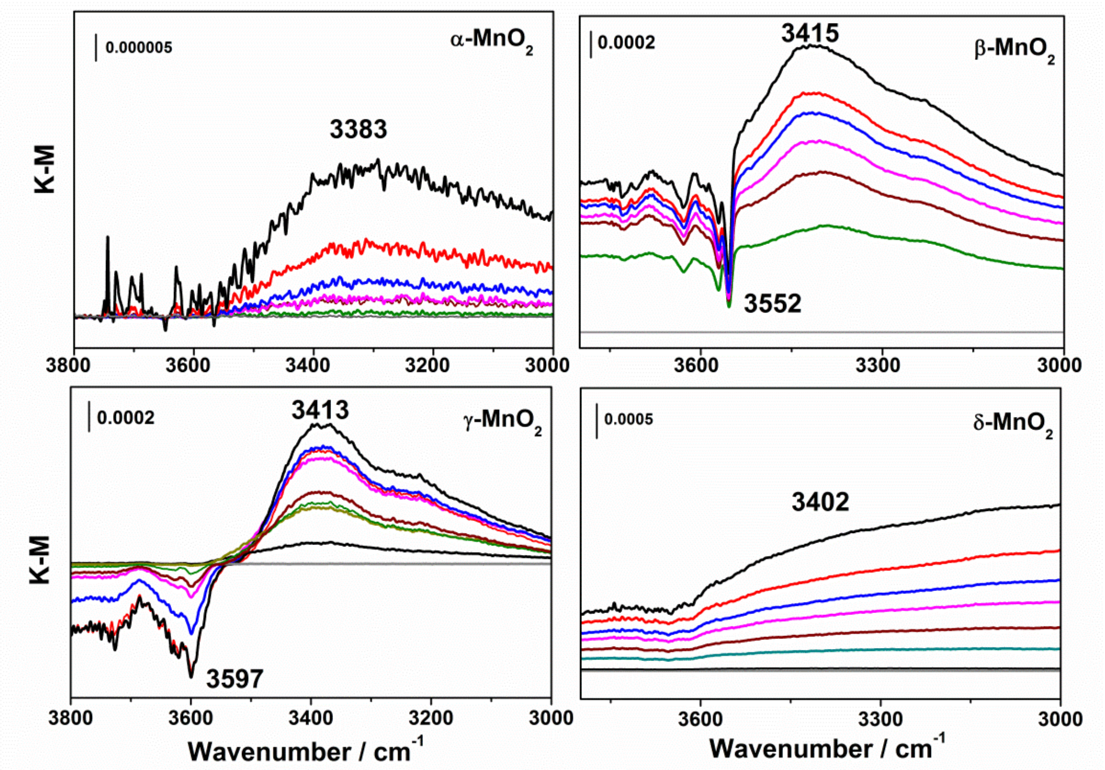

Figure S5. DRIFTS spectra of SO_2 uptake on (a) α-, (b) β-, (c) γ-, (d) δ-MnO_2 sample for 60 min

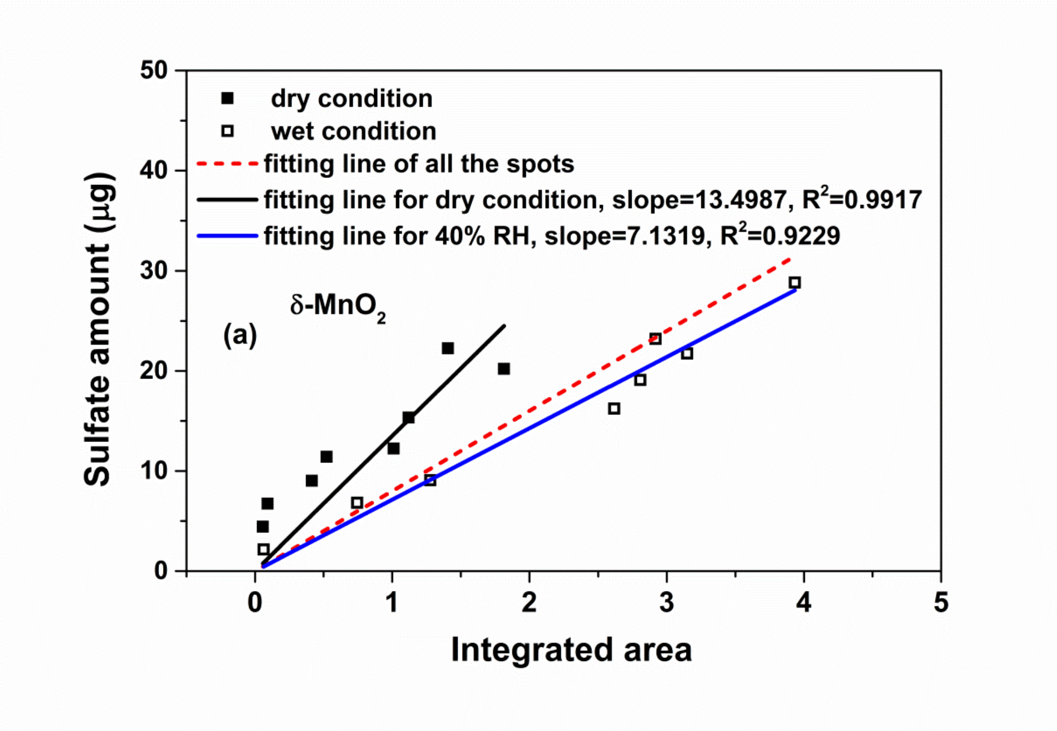

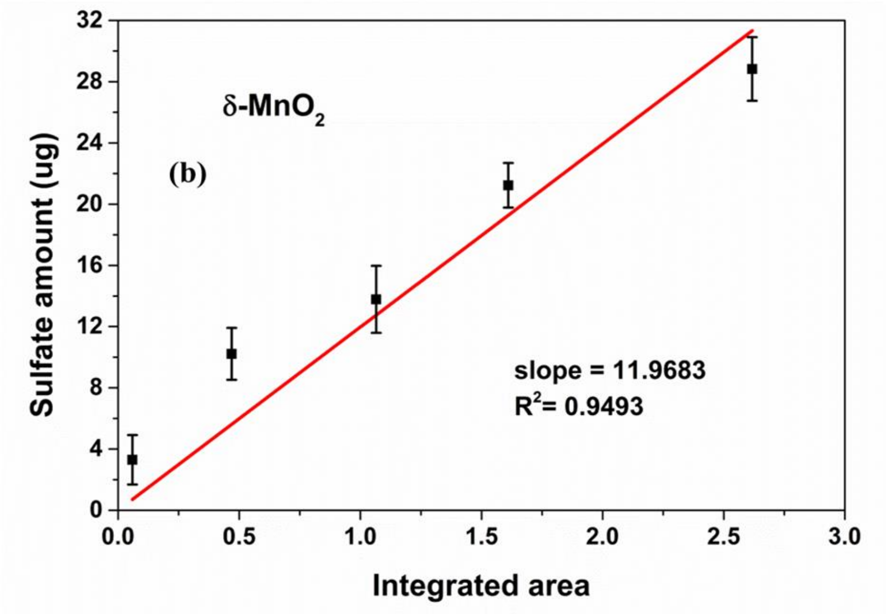

Figure S6. (a) Calibration curves for the integrated absorbance versus the sulfate amount under dry and wet conditions, respectively, and (b) corrected calibration curve compromising the dry and wet conditions for δ-MnO_2.

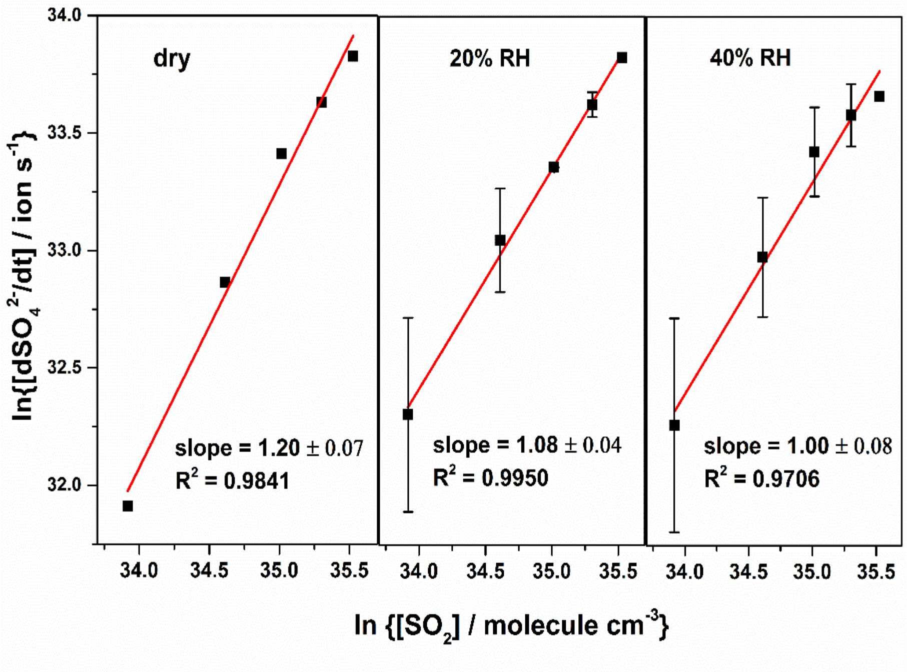

Figure S7. Bilogarithmic plots of the sulfate formation rate versus the SO_2 concentration at different RH for δ-MnO_2.

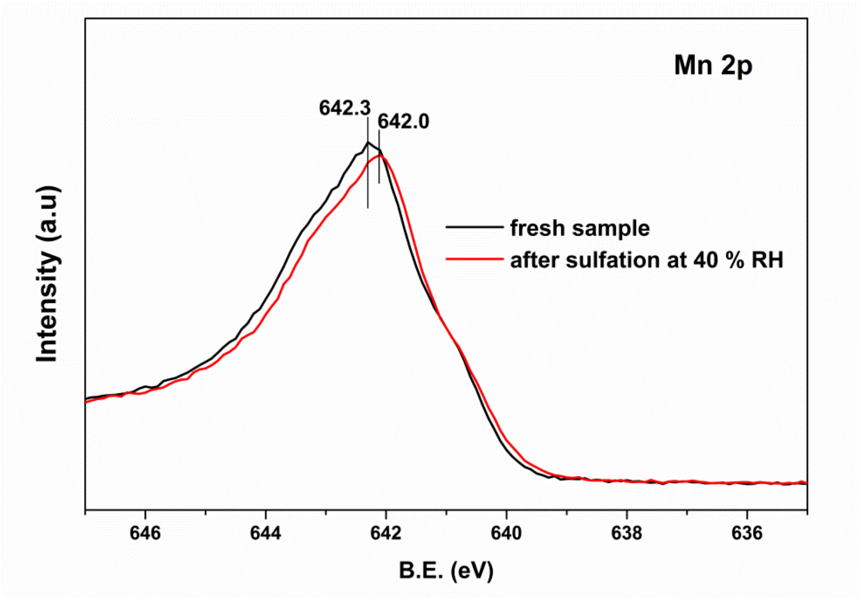

Figure S8. XPS spectra of Mn 2p for fresh and sulfated sample at RH 40%.

1 Zhang, J., Li, Y., Wang, L., Zhang, C. & He, H. Catalytic oxidation of formaldehyde over manganese oxides with different crystal structures. *Catal. Sci. Technol.* **5**, 2305-2313 (2015).

2 Fu, H., Wang, X., Wu, H., Yin, Y. & Chen, J. Heterogeneous Uptake and

Oxidation of SO_2 on Iron Oxides. *J. Phys. Chem. C.* **111**, 6077-6085 (2007).
